# Photoelectron Holographic Atomic Arrangement Imaging of Cleaved Bimetal-intercalated Graphite Superconductor Surface

**DOI:** 10.1038/srep36258

**Published:** 2016-11-04

**Authors:** Fumihiko Matsui, Ritsuko Eguchi, Saki Nishiyama, Masanari Izumi, Eri Uesugi, Hidenori Goto, Tomohiro Matsushita, Kenji Sugita, Hiroshi Daimon, Yuji Hamamoto, Ikutaro Hamada, Yoshitada Morikawa, Yoshihiro Kubozono

**Affiliations:** 1 Graduate School of Materials Science, Nara Institute of Science and Technology, Ikoma, Nara 630-0192, Japan; 2 Research Laboratory for Surface Science, Okayama University, Okayama 700-8530, Japan; 3 Japan Synchrotron Radiation Research Institute, SPring-8, Sayo, Hyogo 679-5198, Japan; 4 Graduate School of Engineering, Osaka University, Suita 565-0871, Japan; 5 International Center for Materials Nanoarchitectonics (WPI-MANA) and Global Research Center for Environmental and Energy based on Nanomaterials Science (GREEN), National Institute for Materials Science (NIMS), 1-1 Namiki Tsukuba 305-0044, Japan; 6 Research Centre of New Functional Materials for Energy Production, Storage and Transport, Okayama University, Okayama 700-8530, Japan

## Abstract

From the C 1*s* and K 2*p* photoelectron holograms, we directly reconstructed atomic images of the cleaved surface of a bimetal-intercalated graphite superconductor, (Ca, K)C_8_, which differed substantially from the expected bulk crystal structure based on x-ray diffraction (XRD) measurements. Graphene atomic images were collected in the in-plane cross sections of the layers 3.3 Å and 5.7 Å above the photoelectron emitter C atom and the stacking structures were determined as *AB*- and *AA*-type, respectively. The intercalant metal atom layer was found between two *AA*-stacked graphenes. The K atomic image revealing 2 × 2 periodicity, occupying every second centre site of C hexagonal columns, was reconstructed, and the Ca 2*p* peak intensity in the photoelectron spectra of (Ca, K)C_8_ from the cleaved surface was less than a few hundredths of the K 2*p* peak intensity. These observations indicated that cleavage preferentially occurs at the KC_8_ layers containing no Ca atoms.

Various graphite intercalation compound (GIC) superconductors have been developed using liquid-metal-alloy (LMA) and vapour-transport (VT) methods^1^,^2^,^3^,^4^,^5^,^6^,^7^,^8^,^9^,^10^,^11^,^12^,^13^. The highest value of the superconducting transition temperature, *T*_c_, for the GIC superconductors at ambient pressure is currently 11.5 K for CaC_6_^6^,^7^. The strong electron-phonon coupling between the electronic state at the interlayer Ca atoms and vertical graphite vibration has been regarded as the origin of this high *T*_c_^8^. Most GIC superconductors have been shown to exhibit increased *T*_c_ under applied pressure^9^,^10^,^11^,^12^. By applying pressure up to 7.5 GPa, the *T*_c_ of CaC_6_ increased to 15.1 K^9^, which is the highest *T*_c_ realized for a GIC superconductor. The increase in *T*_c_ was attributed to the softening of the Ca-Ca phonon under high pressure^10^.

Other than the confirmation of *T*_c_ at 65 mK for BaC_6_^13^, the members of the GIC superconductor family have remained unchanged for the past nine years. However, we recently successfully fabricated a new series of bimetal-intercalated graphite superconductors, Ca_*x*_K_1−*x*_C_*y*_^14^. The *T*_c_ of Ca_*x*_K_1−*x*_C_*y*_ continuously increased to 11.5 K (*x* = 1; CaC_6_) nonlinearly as *x* increased. The *T*_c_ of KC_8_ was only 136 mK, but the introduction of a very small amount of Ca drastically increased this value^14^. The bulk crystal structure was assigned as ‘KC_8_’ type for *x* less than 0.7 and as ‘CaC_6_’ type for *x* = 1 (Supplementary information Fig. S1). K and Ca atoms form 2 × 2 and 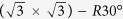 two-dimensional lattices in KC_8_ and CaC_6_, respectively. The vertical arrangements of graphene sheets and K atoms are denoted as ‘*A*α*A*β*A*γ*A*δ’, where ‘*A*’ corresponds to the graphene sheet, and α, β, γ, and δ refer to the sites occupied by metal atoms. Moreover, the arrangements of graphene sheets and Ca atoms are denoted as ‘*A*α*A*β*A*γ’^6^. The distance between graphene sheets, *d*_AA_, in KC_8_ (5.35 Å) is larger than that in CaC_6_ (4.524 Å)^6^, suggesting that graphene layers are more strongly bounded by Ca atom intercalation.

It is essential to directly visualize three-dimensional (3D) atomic arrangements around intercalant atoms to understand the superconducting mechanism, but the local structure around the intercalant atoms of bimetal GIC superconductors remains unknown because the methods available for the local structure analysis were previously limited. Recently, a new holography algorithm, scattering pattern-extraction algorithm (SPEA)-maximum entropy method (MEM), has been developed for atomic arrangement reconstruction^15^,^16^,^17^,^18^. Photoelectron holography is based on photoelectron spectroscopy and diffraction and is used for electronic structure, chemical composition, and atomic arrangement analyses^19^,^20^,^21^,^22^. The 3D image of atomic arrangement around the photoelectron emitter atom can be directly obtained from the element-specific holograms^15^,^16^,^17^,^18^,^22^. Furthermore, the surface phonon is often softened^23^ by the bulk truncation, structure modification, and interaction with the substrate. Surface layer^24^ and monoatomic thin film^25^,^26^ superconductivity have been realized and have attracted substantial fundamental interest. Because of the short probing depth of photoelectrons, photoelectron holography is one of the most effective methods for studying surface and subsurface atomic structures in 3D. Complementally, 3D atomic arrangement images of the bulk can be obtained by fluorescent x-ray holography having longer probing depth^27^,^28^.

Here, we demonstrate the effectiveness of photoelectron holography in the study of the surface of superconducting materials prepared by intercalation with metal atoms. Below, the sample studied here is represented by ‘(Ca, K)C_8_’. In the present study, we successfully measured photoelectron spectra and holography data for superconducting (Ca, K)C_8_, which was recently prepared by our group^14^. The photoelectron spectra of the cleaved surface of (Ca, K)C_8_ showed only C 1*s* and K 2*p* peaks, and the Ca 2*p* peak intensity was less than a few hundredths of that of the K 2*p* peak. We directly obtained the images of C and metal atomic arrangements at the (Ca, K)C_8_ cleaved surface reconstructed from the C 1*s* and K 2*p* photoelectron holograms. The K atoms were found to be located in the centres of the C hexagon columns of the graphene sheets stacked in an *AA* arrangement. Furthermore, the graphite-layered structure without intercalated metal atoms was confirmed to exhibit an *AB* arrangement. Thus, the cleaved surface region of GIC was demonstrated to have a stage 2 structure by photoelectron holography, which differed substantially from the expected bulk (Ca, K)C_8_ crystal structure elucidated from the x-ray diffraction (XRD) measurements. These observations revealed that the cleavage preferentially occurs at the stage-2 type K atom intercalated layers containing no Ca atoms, which is likely the most fragile part of the crystal sample.

## Results

### Characterization of (Ca, K)C_8_

The (Ca, K)C_8_ bimetal GIC lumps were prepared by the LMA technique, in which Ca and K are mixed together with a molar ratio of *x*′:1−*x'* and excess Li. In the present case, the experimental nominal value *x'* was 0.7.

**Figure 1:**
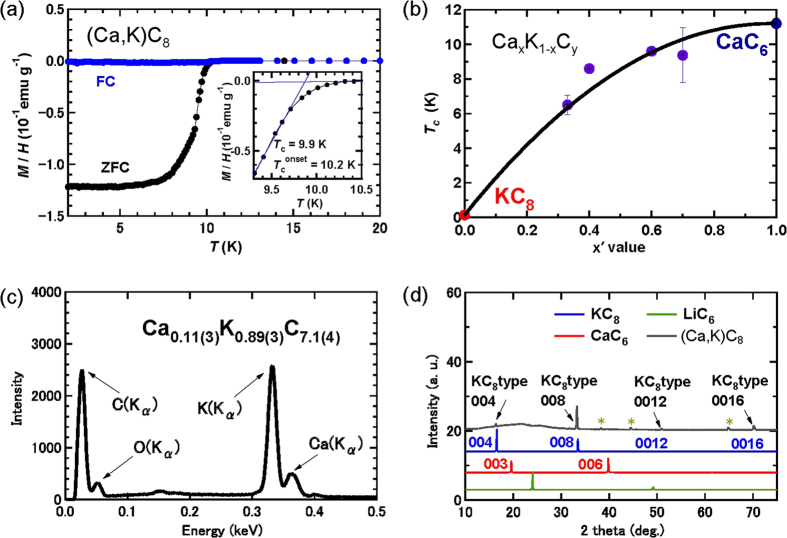
(**a**) *M*/*H vs. T* plots for (Ca, K)C_8_ (ZFC and FC modes) at ambient pressure. (**b**) *x* vs*. T*_*c*_ plots of Ca_x_K_1−x_C_y_. (**c**) EDX spectrum of (Ca, K)C_8._ (**d**) Experimental XRD pattern of (Ca, K)C_8_ and simulated patterns for KC_8_, CaC_6_ and LiC_6_. The peaks labelled ‘*’ in the experimental XRD pattern can be assigned to the Al of the sample holder. The same sample of (Ca, K)C_8_ was used the to obtain the *M*/*H* − *T* plots shown in 1a and the XRD pattern shown in 1c, whereas the sample used for the EDX spectrum shown in 1d is different; however, the nominal *x* value and observed *T*_c_ are the same.

The magnetic susceptibility, χ_g_ (cm^3^ g^−1^), of the present (Ca, K)C_8_ sample is shown in Fig. 1a; 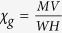, where *MV* (G cm^3^), *W* (g) and *H* (Oe) are the superconducting quantum interference device (SQUID) magnetic moment and weight of the sample and the applied magnetic field, respectively. *T*_c_ was determined from the cross points of two extrapolated lines of the χ_g_–temperature (*T*) plot in the normal and rapid drop regions (see Fig. 1a). The single *T*_c_ at 9.9 K was observed, and the shielding fraction (2.5 K) was nearly 100%, suggesting homogeneous bimetal intercalation throughout most of the bulk region. Figure 1b shows the plot of *T*_c_ versus an experimental nominal value *x *′ for a Ca_*x*_K_1−*x*_C_*y*_ series based on this study and our previous report^14^. *T*_c_ increases continuously with increasing *x *′ in Ca_*x*_K_1−*x*_C_*y*_, suggesting that the Ca and K atoms were intercalated into graphite according to each equilibrium constant.

The energy-dispersive x-ray (EDX) spectroscopy of the ground (Ca, K)C_8_ powder exhibited all peaks of C, K, and Ca atoms shown in Fig. 1c. The EDX showed that the chemical composition was Ca_0.11(3)_K_0.89(3)_C_7.1(4)_. The peak intensity ascribable to Ca atom was quite low compared with the nominal molar ratio (*x*′ = 0.7). This suggests that the equilibrium constant for Ca atom intercalation was much smaller than that of K atom intercalation. However, the fact that the (Ca, K)C_8_ sample with such a small *x* value featured *T*_c_ = 10 K is surprising, as seen in Fig. 1b. This small *x* value (0.11) may be associated with an overestimation of the K concentration ratio because of metallic K residue and the low Ca concentration ratio at the cleaved surface of (Ca, K)C_8_, as suggested from the following photoelectron spectroscopy results.

The out-of-plane XRD pattern (Fig. 1d) of the (Ca, K)C_8_ sample showed the same pattern as the simulated result of KC_8_. Only 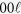 reflections were observed in the XRD pattern, indicating that the present (Ca, K)C_8_ sample was highly oriented along the *c*-axis. The graphene interlayer distance was determined to be 5.38(2) Å, which is nearly equal to one fourth of the lattice constant *c*, 21.40 Å, of KC_8_^3^ with a face-centred orthorhombic (fco) bulk structure: F*ddd* (No. 70).

### Photoelectron spectroscopy and holography of (Ca, K)C_8_

**Figure 2:**
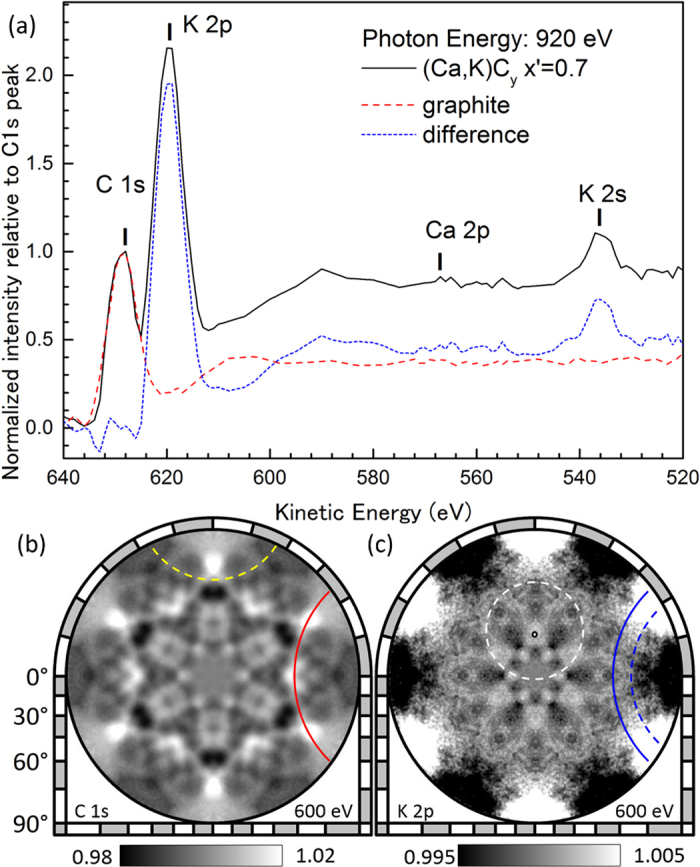
(**a**) X-ray photoelectron spectra. The K 2*p* core-level spectrum was obtained by subtracting the graphite spectrum from that of Ca_0.7_K_0.3_C_y_. (**b**) C 1*s* photoelectron intensity angular distribution (hologram). (**c**) Same as 1b but for K 2*p*.

Prior to the photoemission measurement, we cleaved the (Ca, K)C_8_ lump under an oxygen-free pure Ar gas environment and then placed it in an ultrahigh vacuum chamber without exposing it to atmosphere. Figure 2a shows the photoelectron spectra of (Ca, K)C_8_ and graphite normalized by the C 1*s* peak intensity at a kinetic energy of 628 eV. As indicated by the difference spectrum, the Ca 2*p* peak intensity from the cleaved surface was less than a few hundredths of the K 2*p* peak intensity. Li 1s peak at the binding energy of 56 eV was below the detection limit (not shown). The inelastic mean free path of the photoelectron along the interlayer direction at a kinetic energy of approximately 600 eV was on the order of 2 nm^29^. Therefore, no Li or Ca atoms were present within the surface region of a few graphite layers. Li atoms seem to have been completely replaced by Ca and K atoms during the sample preparation. These results are consistent with the previous conclusion^7^,^8^ that Li metal is removed from the graphite after Ca or K intercalation. Because the EDX result indicated the existence of Ca atoms in the bulk, two scenarios can be suggested: (1) Ca was removed from the surface after cleavage of the (Ca, K)C_8_ lump and (2) a region without Ca was cleaved. Because the vapour pressures of Ca and Ca compounds are sufficiently low at room temperature, the first scenario is excluded. Because graphene layers are more strongly bounded by Ca than K intercalation, it is reasonable to conclude that cleavage occurs selectively at the layers without Ca atoms.

Full-hemisphere photoelectron holograms were recorded for C 1*s* and K 2*p,* as shown in Fig. 2b,c (Supplementary information Fig. S2). The holograms are represented in a stereo projection so that diffraction rings appear as circles^29^. Yellow-dashed and red-solid arcs in the C 1*s* hologram correspond to the diffraction rings around the directions along and perpendicular to the shortest CC bonds of graphite, respectively. The diffraction feature resembling the C 1*s* hologram also appeared as a background in the measured K 2*p* hologram because of the energy-loss components of C 1*s* photoelectrons^30^. The intrinsic C 1*s* and K 2*p* holograms (*I*_C_ and *I*_K_) shown in Fig. 2b,c, respectively, were obtained by solving a simultaneous equation (Supplementary information Fig. S3). The raw C 1*s* and K 2*p* holograms consisted of 0.95*I*_C_ + 0.05*I*_K_ and 0.475*I*_C_ + 0.525*I*_K_, respectively. The coefficients were determined by considering the graphite spectrum shown in Fig. 2a and the residual C 1*s* background pattern in the K 2*p* hologram. The direction of the forward focusing peak (FFP) and the diffraction ring indicated by white marks in the K 2*p* hologram correspond to the directions of the nearest neighbouring C atom above. The blue arcs are attributed to the 2 × 2-ordered K atomic arrangement, as discussed below.

### 3D structure images of (Ca, K)C_8_

**Figure 3:**
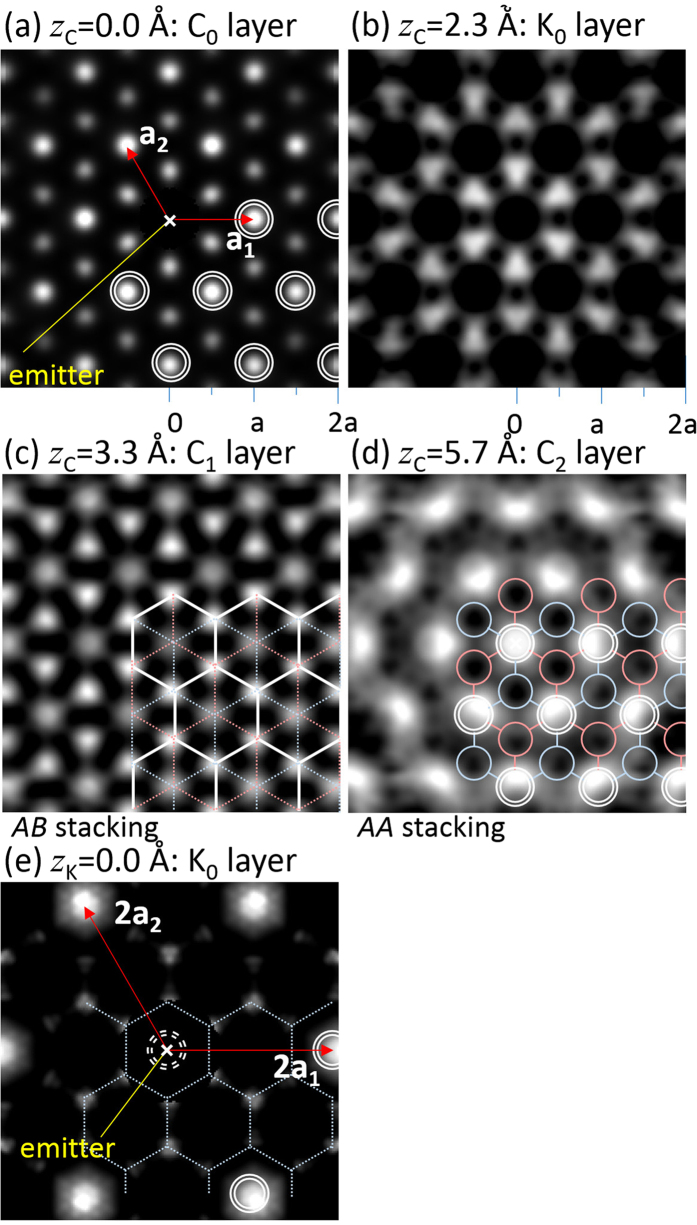
(**a**) Atomic arrangement reconstructed from the C 1*s* photoelectron hologram. The cross section at the layer including the photoelectron emitter C atom is indicated. (**b**–**d**) Same as 1a but for the layers above the emitter atom by 2.3, 3.3, and 5.7 Å, respectively. (**e**) Same as 1a but from the K 2*p* photoelectron hologram.

The atomic images reconstructed from the C 1*s* and K 2*p* photoelectron holograms are summarized in Fig. 3 (Supplementary information Fig. S4). The holography transformation algorithm SPEA-MEM was applied to the photoelectron holograms, and the 3D structure images around the photoelectron emitter C and K atoms were obtained. For the reconstruction from the C 1*s* hologram, the C atom potential was used for the scatterer, whereas for the K 2*p* hologram, the K atom potential was used. Therefore, the interatomic distances from the C atom to the K atom and from the K atom to the C atom were underestimated and overestimated, respectively. Figure 3a shows the atomic arrangements reconstructed from the C 1*s* photoelectron hologram. The cross section at the layer including the photoelectron emitter C atom is indicated. The emitter atom is marked by the ‘x’ in the centre because it does not appear in the reconstructed image. We introduced a translational symmetry mixing operation and examined the reconstructed image by varying the in-plane lattice constant. An in-plane lattice constant of 2.50 ± 0.05 Å resulted in the best converged atomic images. Figure 3b–d were also reconstructed from the C 1*s* photoelectron hologram but for the layers above the emitter atom by 2.3, 3.3, and 5.7 Å, respectively. Note that the atomic image contrast at each triangular lattice point was different. The first and second neighbouring atom images around the centre appeared as dark and bright spots, respectively, in Fig. 3a, whereas the second neighbouring atom images appeared as bright spots in Fig. 3c. Furthermore, the bright atomic images formed hexagonal and triangular lattices in Fig. 3b,d.

**Figure 4:**
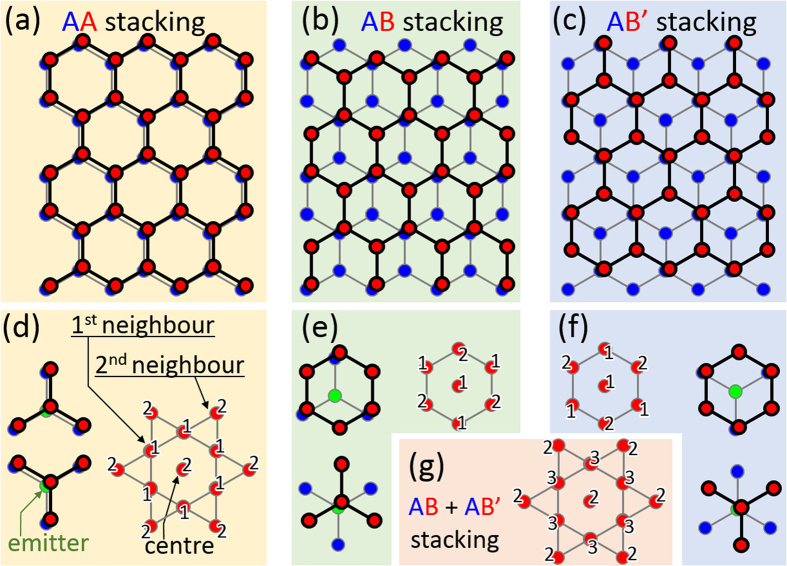
Atomic configuration models of (**a**) *AA*, (**b**) *AB*, and (**c**) *AB*′ bilayer stacking structures. Graphene clusters can be stacked on top of the *A* graphene layer in three different ways. (**f**–**g**) The schematic structure model in each stacking geometry and the expected signal intensity ratio at the centre; the first- and the second-neighbouring atoms in the reconstructed real space images.

There are various types of graphene layer stacking, as shown in Fig. 4. The “B” layer can be stacked on the “A” layer with a translational shift equal to the CC bond length in the CC bond direction as shown in Fig. 4b, or the “B” layer can be stacked with a translational shift in the opposite direction, as shown in Fig. 4c. The stacking registry can be determined from the modulation of the atomic image contrast as shown in Fig. 4d–g. For the layer stacked in the *AA* (Fig. 4d) and *AB* + *AB*′ (Fig. 4g) sequences, bright spots form triangular and hexagonal lattices, respectively. The contrast modulation of the C layer at *z*_C_ = 3.3 Å (Fig. 3c) is a strong indication that the graphite structure is stacked in the *AB* pattern. Since the intensity in the blue/red circle in Fig. 3d was smaller than in the white circle, we concluded that atomic arrangement at the C layer at 5.7 Å is not AB type. Although the first neighbouring atoms were not well reproduced, the photoelectron diffraction simulation analysis described in the next section fitted well, the C layer at 5.7 Å (Fig. 3d) is attributed to a graphene layer stacked in the *AA* pattern.

The atomic images in Fig. 3b are located at the centre of C hexagon rings. These spots are attributed to the intercalated K atoms between two *AA*-stacked graphene layers. The metal–graphene interlayer distance in bulk KC_8_ crystals is 2.68 Å. The underestimation of the K layer height as 2.3 Å was because of the C atom potential used for the holographic reconstruction^17^. Figure 3e is the atomic arrangement at the layer including the photoelectron emitter K atom reconstructed from the K 2*p* photoelectron hologram. For the K atomic image reconstruction, we examined various periodic structure such as 1 × 1, 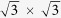 and incommensurate lattices. As a result, we found that the 2 × 2 translational symmetry gave the best converged atomic images. K atoms arranged in 2 × 2 periodicity were imaged.

C atom sites in the graphene layer have a mirror symmetric relation and they are identical in terms of energy. Therefore, C 1s XPS peak for those two sites appear at the same binding energy and cannot be separated using chemical shift information. To separate the two different crystal lattice images from those two types of C atom sites superimposed in the reconstructed images, we defined the following filter patterns F_+_(**r**) and F_−_(**r**). Sum of F+ and F− is unity.
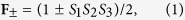

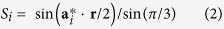


**Figure 5:**
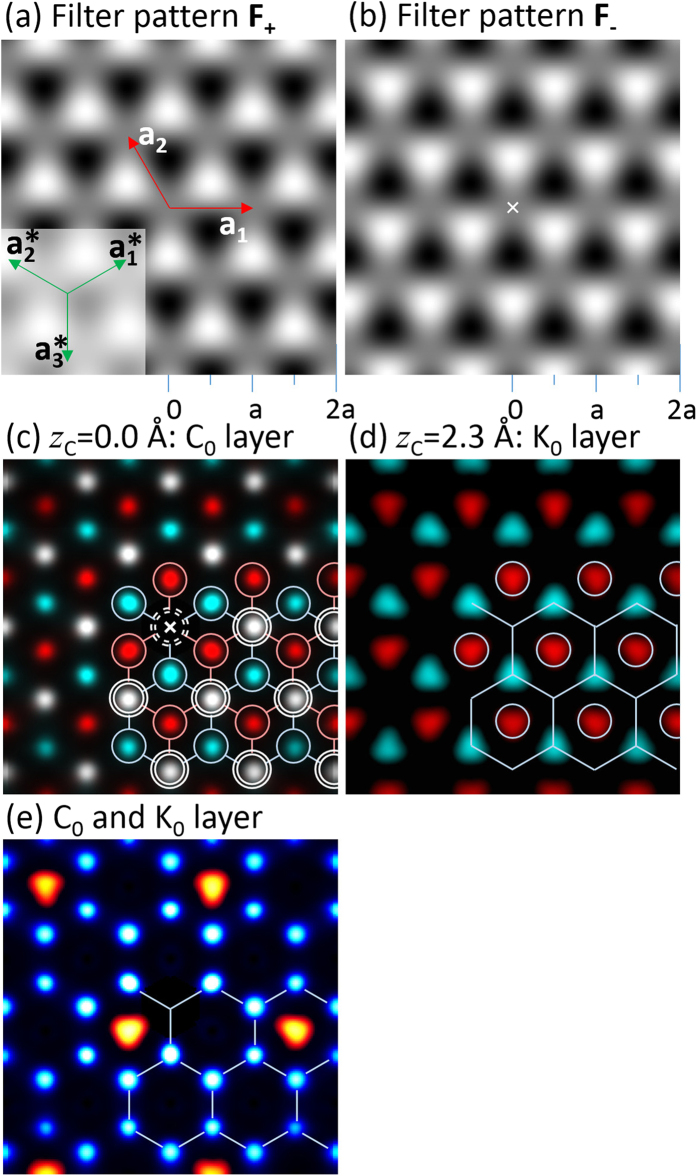
(**a**,**b**) Filter patterns. See the text for the definition. (**c**,**d**) C and K atom images coloured differently for two different lattices obtained by multiplying images Fig. 3a,b, respectively, with the filter pattern shown in 5a,b. (**e**) Atomic arrangement image of C and K layers obtained from same type of lattice images for C and K in 5c,d.

Figure 5a,b shows the image of the filter pattern F_+_(**r**) and F_−_(**r**), respectively. 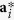 is a reciprocal lattice vector, as indicated in the inset of Fig. 5a. The triangular lattice point corresponding to the C atom site has a value of 0, 0.5, or 1. By multiplying the filter patterns by the images shown in Fig. 3a,b, we obtained two different lattice images shown coloured in blue (F_+_) and red (F_-_) in Fig. 5c,d, respectively. The atomic images at the overlapping sites appeared as white spots. Note that in Fig. 5c we obtained a hexagonal lattice, while in Fig. 3e, we obtained K 2 × 2 lattice. This is because the K lattice in Fig. 5c is seen from the different kind of C emitter atoms, while in Fig. 3e, the emitter is single kind of K atom. By combining one type of lattice image of the C and K layers, we finally obtained the GIC atomic arrangement image shown in Fig. 5e.

### Local structure evaluation by theoretical approaches. 

**Figure 6:**
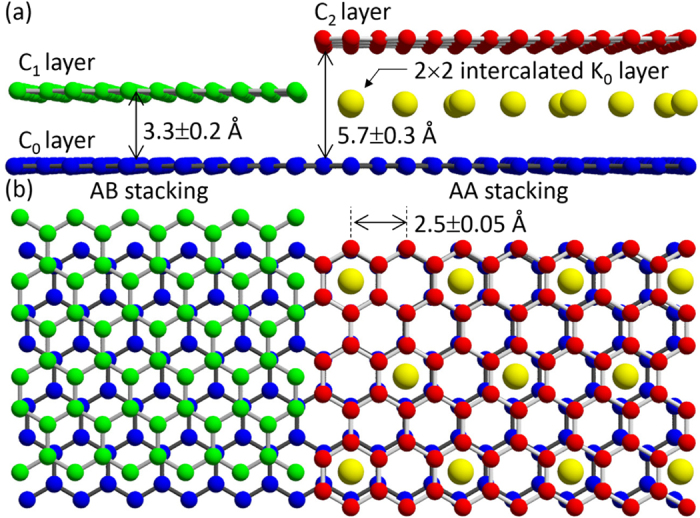
Schematic diagrams of (**a**) the vertical cross section and (**b**) top view of GIC.

The detailed local structure around the intercalant atoms was revealed as summarized in Fig. 6a,b. The error bars for the interlayer distances for *AB* and *AA* stacking were determined from the distribution of vertical atomic images reconstructed by the holography algorithm. We also performed density functional theory (DFT) calculations. Table 1 summarizes the bulk lattice constants^3^,^31^ and the present data for the cleaved surface compared with the values derived from the DFT calculation. As seen in Table 1, by employing recently proposed van der Waals density functional by Hamada, rev-vdW-DF2^32^ along with Wu and Gygi’s separable form of the vdW kernel^33^,^34^, the in-plane as well as inter-layer lattice constants of graphite and bulk KC_8_ can be very well reproduced. Note that the in-plane and interlayer lattice parameters are larger than those of graphite and bulk KC_8_. The former is attributed to the charge transfer from a metal intercalant atom to the graphite lattice; this is supported by the calculated value. The surface interlayer lattice expansion is attributed to the surface relaxation effect. Although the present experimental value derived from photoelectron holography and the calculated value were within the error, the slight difference between these two values could relate to the effects of excess K and oxygen contamination on the surface.

**Figure 7:**
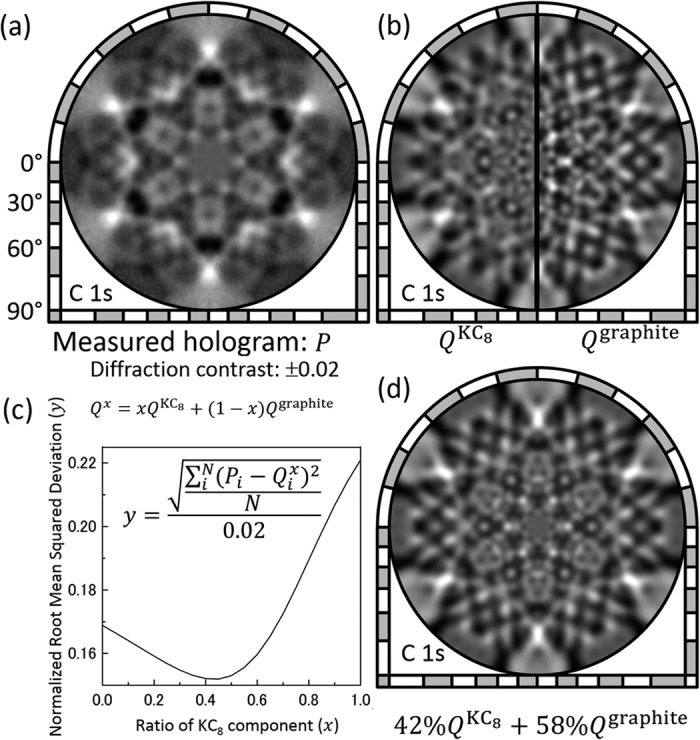
(**a**) Measured C 1*s* photoelectron hologram *P*. (**b**) Simulated C 1*s* photoelectron holograms *Q*^KC^_8_ and *Q*^graphite^ for KC_8_ and graphite, respectively. (**c**) Root-mean-squared deviation of the measured and simulated C 1*s* holograms averaged over *N* pixels and normalized by the diffraction contrast of *P*. (**d**) Linear combination of the two holograms *Q*^KC^_8_ and *Q*^graphite^ with a ratio of 42% to 58%.

Based on these structural parameters obtained by photoelectron holography, we simulated C 1*s* holograms *Q* for the KC_8_ and graphite surfaces as shown in Fig. 7b. Using these two simulated holograms as a basis set, the measured C 1*s* hologram *P* (Fig. 7a) was fitted. Figure 7c shows the root-mean-squared deviations of the measured and simulated C 1*s* holograms averaged over all pixels and normalized by the diffraction contrast. The linear combination pattern of *Q*^KC^_8_ and *Q*^graphite^ at a ratio of 42% to 58% most resembled *P* with the lowest root-mean-square deviation. Thus, indicates K intercalation occurs at stage 2. These results also support the conclusion that the cleavage takes place selectively at the layers without Ca atoms and at stage 2-type regions where the graphite layers are weakly bounded.

## Discussion

The most important result from the present study is that the cleaved surface does not always represent the averaged bulk structure. The structure and composition of the surface and the bulk can be substantially different, especially in a layered system. Special attention must be paid when using surface-sensitive methods, such as photoelectron spectroscopy and scanning probe microscopy. The structure of the cleaved surface region of GIC was successfully clarified by photoelectron holography and was found to differ substantially from the expected bulk (Ca, K)C_8_ crystal structure determined by XRD. These observations revealed that the cleavage preferentially occurred at the KC_8_ layers containing no Ca atoms. The K atoms were found to be located in the centre of C hexagons of the graphene sheets stacked in the *AA* arrangement with an interlayer spacing of 5.7 Å. In addition, the graphite-layered structure was confirmed to exhibit *AB* stacking and an interlayer spacing of 3.3 Å. This result supports the existence of stage 2-type graphite layers without intercalated metal atoms and the conclusion that cleavage occurs selectively at the weakly bounded layers. Photoelectron holography is a unique method for the 3D visualization of local atomic structures at the surface region.

From the out-of-plane XRD analysis, the graphene interlayer distance was determined to be similar to that of KC_8_, suggesting that the K atoms, which have a larger ion radius, play dominant roles in determining the vertical structure. However, EDX measurements revealed that Ca atoms efficiently contribute to the realization of relatively high *T*_c_ despite the low Ca concentration. The observation of the K dominant structure at the cleaved surface implies that Ca atoms are dispersed in the bulk and likely form high *T*_c_ domains.

## Methods

### Sample preparation and characterizations

The samples of (Ca, K)C_8_ were prepared using the LMA method^6^. The stoichiometric ratio of Ca and K was established with an excess amount of Li in the vessel, which was heated at 573 K. The Ca/K/Li alloy melted at 573 K. The kish graphite was immersed into the melted Ca/K/Li alloy in the vessel for one week. The superconductivity of the (Ca, K)C_8_ sample was checked by recording the DC magnetic susceptibility (*M*/*H*) with a SQUID magnetometer (Quantum Design MPMS2). The XRD pattern of the sample was measured at 297 K using an XRD instrument (Rigaku, Smart Lab-Pro) with a Cu Kα source (*λ* = 1.5418 Å).

### Measurements of photoelectron spectra and holograms

The C 1*s* and K 2*p* photoelectron intensity angular distributions (holograms) from the GIC cleaved surface were measured using a display-type spherical mirror analyser (DIANA)^35^,^36^ at the circularly polarized soft x-ray beamline BL25SU of SPring-8, Japan^37^. The acceptance sold angle of the analyser was 1π steradian (±60°). The x-ray incident direction was set along the surface normal axis. The energy of the electrons emitted from the sample was analysed, and their angular distributions were projected onto the fluorescent screen. A full-hemispherical photoelectron hologram was obtained by rotating the sample azimuthal angle by 360°^36^. We measured for several samples (typically 3 × 2 mm^2^ in size). We surveyed the spatial distribution of the sample and confirmed that when the target area (1 × 1 mm^2^) were larger than the beam size (diameter: several 10 μm were uniform, we get clear diffraction patterns. The holography transformation code SPEA-MEM was used for the real-space atomic arrangement image reconstruction. One-dimensional polar angle photoelectron intensity profiles 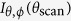 were derived for every direction, 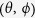, from the measured holograms. Oscillatory structures resulting from the diffraction rings appeared in the neighbouring atom directions but not in the other directions where no atoms existed. We then calculated the scattering pattern matrix at a kinetic energy of 600 eV. One scattering pattern matrix consisted of a set of fundamental diffraction patterns for polar angles 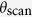 of 0 to 180° and atomic distances of 0.1 to 1.1 nm. One-dimensional profiles 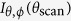 were fitted using the maximum entropy method with these fundamental diffraction patterns corresponding to the positions of 10-pm cubic voxels within 0.7 nm of the emitter atom.

### Calculation details

DFT simulations were conducted using the Simulation Tool for Atom TEchnology (STATE-Senri) code. A recently proposed van der Waals density functional by Hamada, rev-vdW-DF2^32^ was implemented with Wu and Gygi’s separable form of the vdW kernel^33^,^34^. The inner electrons were replaced by ultrasoft-pseudopotentials and a plane wave basis set with cutoff energies of 36 Ry for wave functions and 400 Ry for charge density is employed. Methfessel and Paxton scheme of the Fermi-level smearing (σ = 0.054 eV) was used to improve the convergence.

## Additional Information

**How to cite this article**: Matsui, F. *et al*. Photoelectron Holographic Atomic Arrangement Imaging of Cleaved Bimetal-intercalated Graphite Superconductor Surface. *Sci. Rep.* **6**, 36258; doi: 10.1038/srep36258 (2016).

**Publisher’s note:** Springer Nature remains neutral with regard to jurisdictional claims in published maps and institutional affiliations.

## Supplementary Material

Supplementary Information

## Figures and Tables

**Table 1:** Experimental and DFT calculation results for the estimation of the in-plane lattice constant *a* and the interlayer distances *d*_*AA*_.

		bulk (Å)		surface (Å)			
		XRD*	calc.	calc.		Exp. This report	
Graphite	*a*	2.456	2.456 (0.0%)				1 × 1
	*d*_*AA*_	3.348	3.356 (+0.2%)	3.348	−0.2%	3.3 ± 0.2	C_0_ – C_1_
KC_8_	*a*	4.96	4.974 (+0.3%)			5.0 ± 0.1	2 × 2
	*d*_*AA*_	5.35	5.362 (+0.2%)	5.366	+0.1%	5.7 ± 0.3	C_0_ – C_2_

^*^Values from reference [31] for graphite and [3] for KC_8_.
