# Supplementary material for: Photoelectron Holographic Atomic Arrangement Imaging of Cleaved Bimetal-intercalated Graphite Superconductor Surface
Source: Sci Rep. 2016 Nov 4;6:36258. doi: 10.1038/srep36258 (PMC5095891; doi:10.1038/srep36258)
Supplement: Supplementary Information [file srep36258-s1.pdf]

Electronic supplementary information

Photoelectron Holographic Atomic Arrangement Imaging of Cleaved Bimetal-intercalated Graphite Superconductor Surface

Fumihiko Matsui[1,*], Ritsuko Eguchi[2], Saki Nishiyama[2], Masanari Izumi[2], Eri Uesugi[2], Hidenori Goto[2], Tomohiro Matsushita[3], Kenji Sugita[1], Hiroshi Daimon[1], Yuji Hamamoto[5], Ikutaro Hamada[6], Yoshitada Morikawa[5], Yoshihiro Kubozono[2,4]

[1] Graduate School of Materials Science, Nara Institute of Science and Technology, Ikoma, Nara 630-0192, Japan

[2] Research Laboratory for Surface Science, Okayama University, Okayama 700-8530, Japan

[3] Japan Synchrotron Radiation Research Institute, SPring-8, Sayo, Hyogo 679-5198

[4] Research Centre of New Functional Materials for Energy Production, Storage and Transport, Okayama University, Okayama 700-8530, Japan

[5] Graduate School of Engineering, Osaka University, Suita 565-0871, Japan

[6] International Center for Materials Nanoarchitectonics (WPI-MANA) and Global Research Center for Environmental and Energy based on Nanomaterials Science (GREEN), National Institute for Materials Science (NIMS), 1-1 Namiki Tsukuba 305-0044, Japan.

*Correspondence to matui@ms.naist.jp

Bulk structures

The bulk crystal structure of KC_8 refers to the face-centered orthorhombic structure (fco) and the space group is *F*ddd. The arrangements of metal atom and graphene sheet is shown as 'AαAβAγAδ'; 'A' corresponds to graphene sheet, while α, β, γ and δ refer to the sites occupied by metal atoms. The lattice constants, *a*, *b* and *c*, of KC_8 are 4.92, 8.52 and 21.4 Å, respectively.

The bulk crystal structure of CaC_6 refers to the trigonal structure and the space group is *R*-3m. The arrangements of metal atom and graphene sheet is shown as 'AαAβAγ'. The lattice constants, *a* and *c*, of KC_8 are 4.434 and 13.572 Å, respectively.

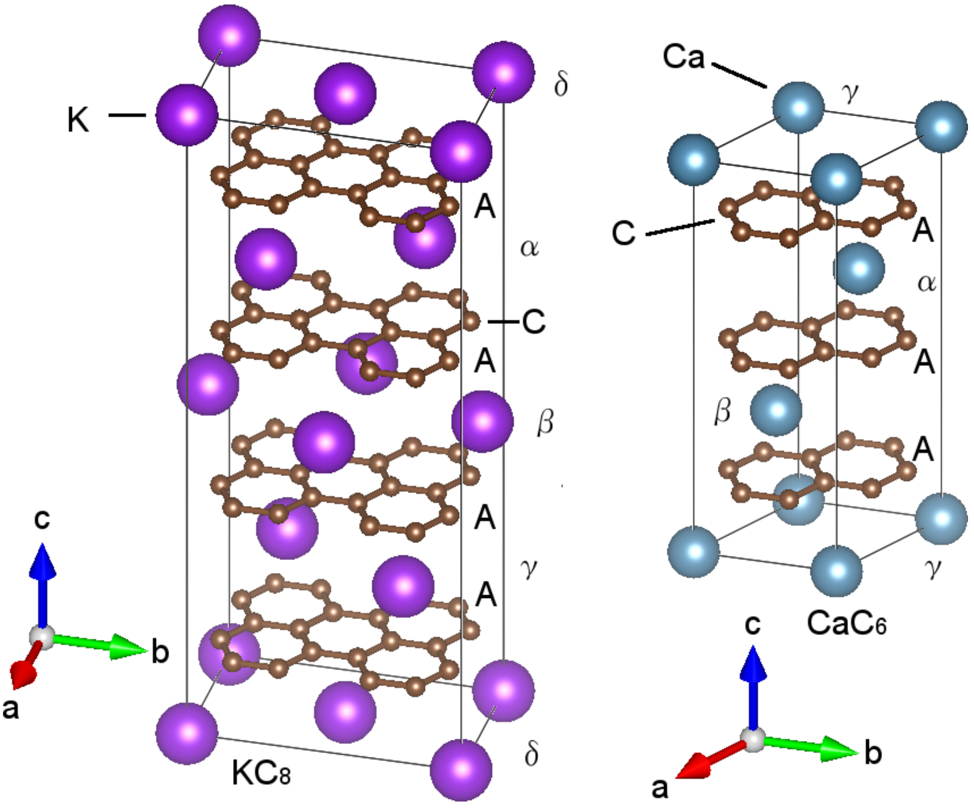

Figure S1: Bulk structure model of KC_8 and CaC_6.

2π-steradian photoelectron intensity angular distribution

Soft X-ray was incident from surface normal direction. Sample azimuthal orientation was varied from 0° to 360° by 9° step. Dwell time was 3 min for each PIAD acquisition. Total acquisition time was about 2 hours. However, this pattern contains artifacts due to detection inhomogeneity. Transmission function of the two-dimensional detector, *i.e.*, inhomogeneous detection efficiency distribution was obtained by simply averaging all the measured data. Position of photelectron diffraction pattern on the screen varies as the sample orientation changes, but the position of artifact pattern due to inhomogeneous detection efficiency does not change its position. By normalizing measured patterns using the averaged pattern, inhomogeneous detection efficiency was removed as shown in Fig. S2. They are assembled to one 2π-steradian PIAD. Note that the polar angle dependence information, especially the diffraction pattern at [001] direction (center of the pattern) disappeared

due to this normalization process. Six-fold symmetry operation was applied to the obtained 2π-steradian PIAD patterns.

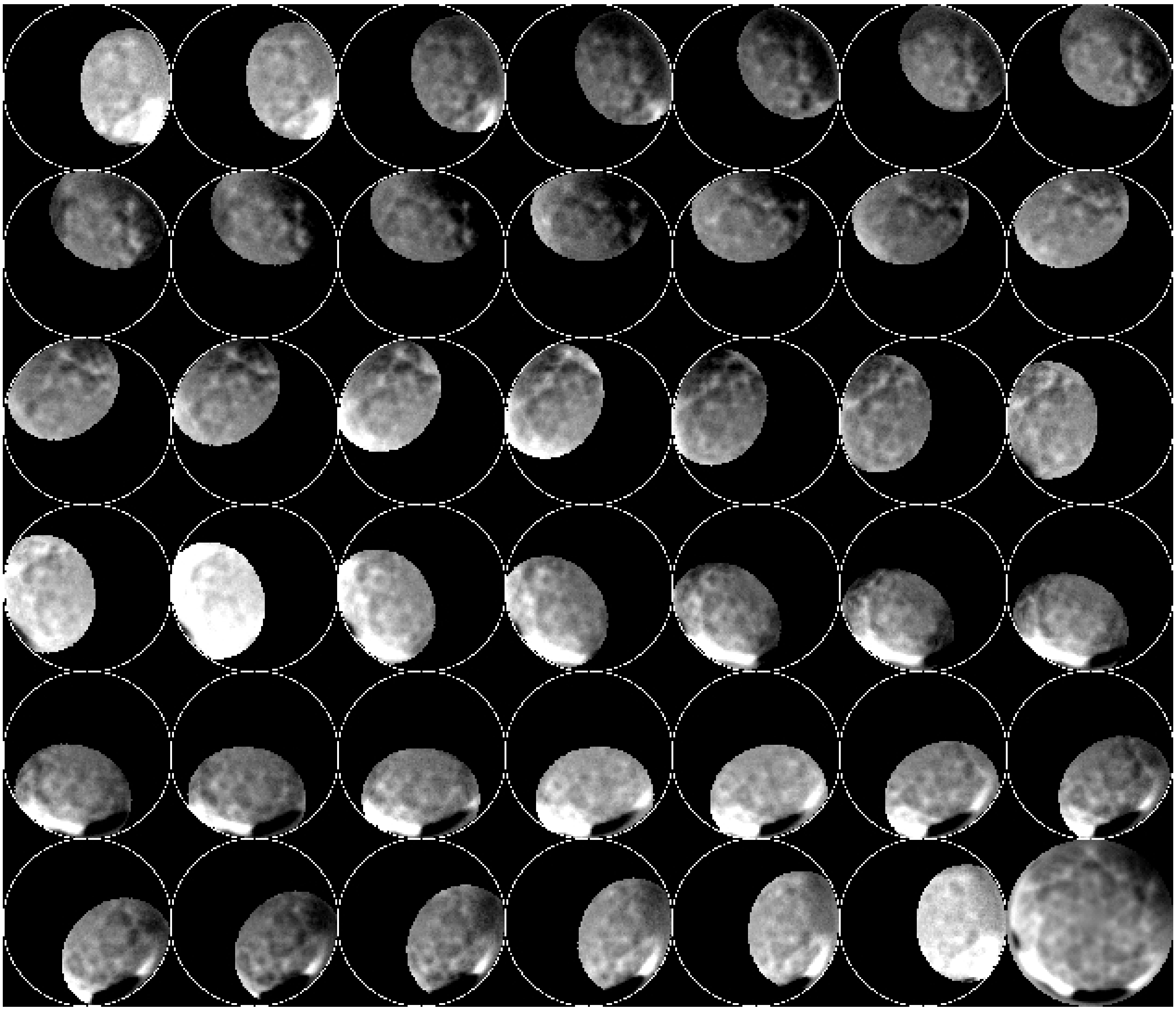

Figure S2: A series of 41 C 1s photoelectron intensity angular distributions (PIAD) normalized by transmission function of the detector and plotted using the azimuthal equidistance projection. The center and periphery of the circle correspond to the surface normal and horizon directions, respectively. They are combined into 2π-steradian C 1s PIAD pattern shown in right bottom.

Photoelectron pattern simultaneous equation

Figure S3a and b show the measured full-hemisphere photoelectron holograms for C 1*s* (P_C) and K 2*p* (P_K), respectively. The diffraction feature resembling the C 1*s* hologram also appeared as a background in the measured K 2*p* hologram because of the energy-loss components of C 1*s* photoelectrons. The K 2*p* peak was partially overlapping with the C 1*s* peak. Therefore the measured photoelectron holograms C 1*s* (P_C) and K 2*p* (P_K) can be expressed as the linear combination of the intrinsic C 1*s* (I_C) and K 2*p* (I_K) holograms.

$$\begin{pmatrix} P_C \\ P_K \end{pmatrix} = \begin{pmatrix} 1-m & m \\ n & 1-n \end{pmatrix} \begin{pmatrix} I_C \\ I_K \end{pmatrix}$$

The intrinsic C 1s and K 2p holograms (I_C and I_K) shown in Fig. 2b and 2c, respectively, were obtained by solving a simultaneous equation.

$$\begin{pmatrix} I_C \\ I_K \end{pmatrix} = \begin{pmatrix} 1-m & m \\ n & 1-n \end{pmatrix}^{-1} \begin{pmatrix} P_C \\ P_K \end{pmatrix} = \frac{1}{1-m-n} \begin{pmatrix} 1-n & -m \\ -n & 1-m \end{pmatrix} \begin{pmatrix} P_C \\ P_K \end{pmatrix}$$

The coefficients m was determined to be 0.05 by considering the graphite spectrum shown in Fig. 2a. The coefficients n was determined to be 0.475 inspecting the possible combinations of m and n and judging from the residual C 1s background pattern in the K 2p hologram as shown in Fig.S3c. The raw C 1s and K 2p holograms (P_C and P_K) consisted of $0.95I_C + 0.05I_K$ and $0.475I_C + 0.525I_K$, respectively.

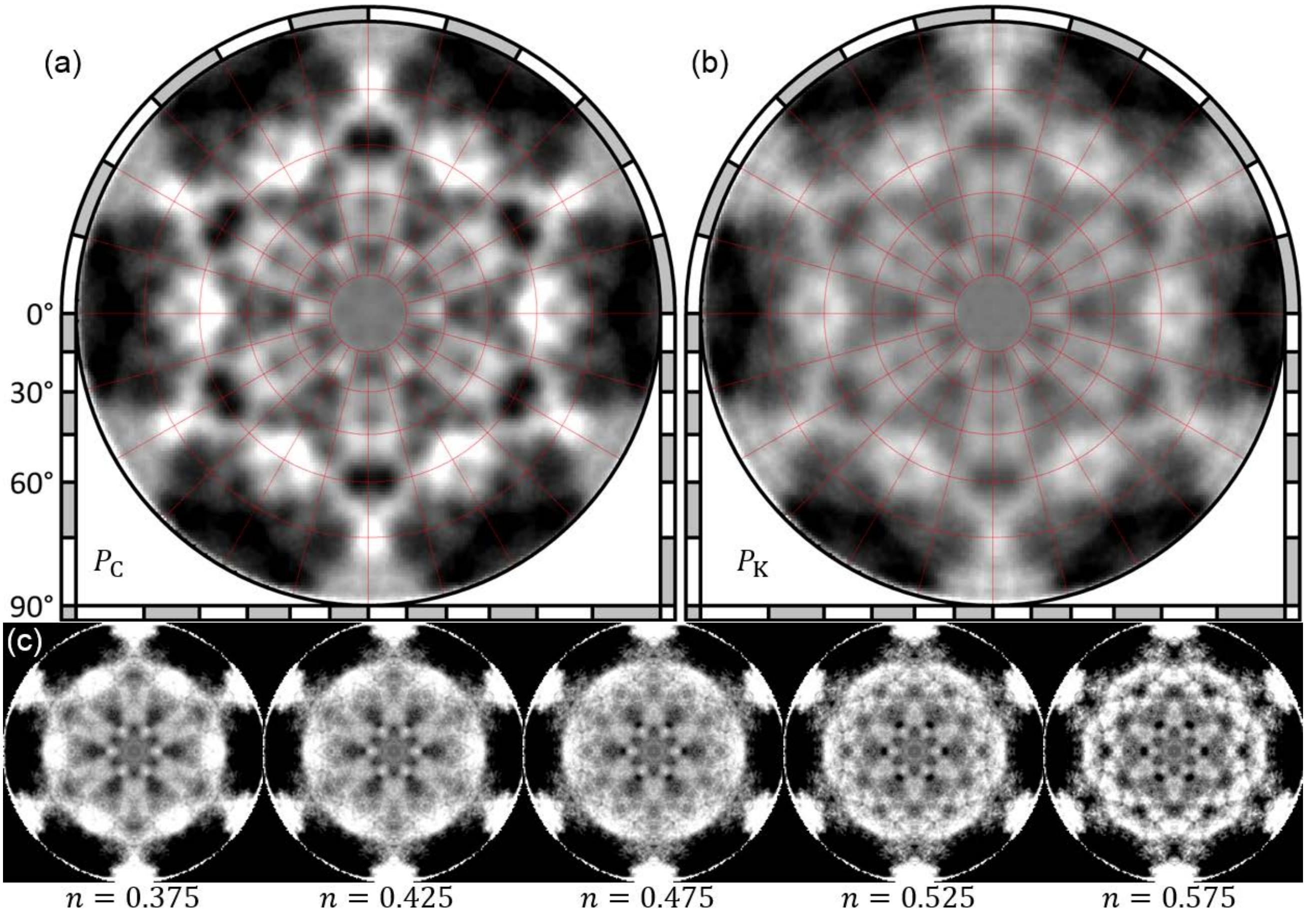

Figure S3: (a) and (b) The measured full-hemisphere photoelectron holograms for C 1s (P_C) and K 2p (P_K), respectively. (c) A series of linear combination of P_C and P_K patterns with parameter m fixed at 0.05 and n varied as indicated value.

Scattering pattern matrix

Photoelectron holography is a method for atomic structural analysis. The first reconstruction algorithm proposed by Barton in 1988 was based on the Fourier transformation; however, a clear

atomic image could not be obtained by this algorithm because the effect of the phase shift of the scattering process was neglected. In addition, a conjugate image, which is a virtual image located on the point symmetric position, appears. In order to solve the problem of the conjugate image, using a series of photoelectron hologram with different kinetic energy was effective. Although this enabled the conjugate image problem to be solved, the effect of the phase shift could not be solved. The typical phase shift problem is the forward focusing effect, a phenomenon in which electrons are focused toward the direction of the scatterer atom. The forward focusing peak caused image aberrations in the reconstructed atomic image.

On the other hand, we developed a reconstruction algorithm, SPEA-MEM, which does not utilize the Fourier transformation. SPEA-MEM is a holography transformation code based on scattering pattern extraction algorithm and maximum entropy method. This algorithm solves the problem of the phase shift and the conjugate image, and the atomic arrangement can be obtained without using the multi-energy format. We succeeded in reconstructing a three-dimensional atomic arrangement from a single-energy hologram with a resolution of 0.02 nm. This algorithm enables the atomic arrangement to be measured from a photoelectron hologram using an X-ray tube or an Auger electron hologram.

SPEA-MEM was used for the real-space atomic arrangement image reconstruction for the present study. One-dimensional polar angle photoelectron intensity profiles $I_{\theta,\phi}(\theta_{\mathrm{scan}})$ were derived for every direction, (θ,ϕ), from the measured holograms. Oscillatory structures resulting from the diffraction rings appeared in the neighbouring atom directions but not in the other directions where no atoms existed. We then calculated the scattering pattern matrix at a kinetic energy of 600 eV. Figure S3 shows the example of scattering pattern matrix for C 1s core level emission consisted of a set of fundamental diffraction patterns for polar angles θ_{scan} of 0 to 180° (vertical abscissa) and C to C atomic distances of 0.1 to 1.1 nm (horizontal abscissa). One-dimensional profiles $I_{\theta,\phi}(\theta_{\mathrm{scan}})$ were fitted using the maximum entropy method with these fundamental diffraction patterns corresponding to the positions of 10-pm cubic voxels within 0.7 nm of the emitter atom.

The three-dimensional voxels that represent atomic distributions can be linearly converted to the photoelectron hologram pixel using the scattering pattern matrix. When photoelectron hologram image is given, the solution of this linear equation may give the atomic distribution. It is, however, difficult to solve because the amount of the voxels is quite larger than that of the hologram pixels. To solve this, SPEA-MEM has been adopted a fitting-based algorithm using the maximum entropy method.

The further details of SPEA-MEM algorithm were explained in the published references (Matsushita et al. J. Electron Spectrosc. Relat. Phenom. **178-179** (2010) 195 and **195** (2014) 365). The latest version of this code is available from the following web site; https://ja.osdn.net/projects/tmcoca/.

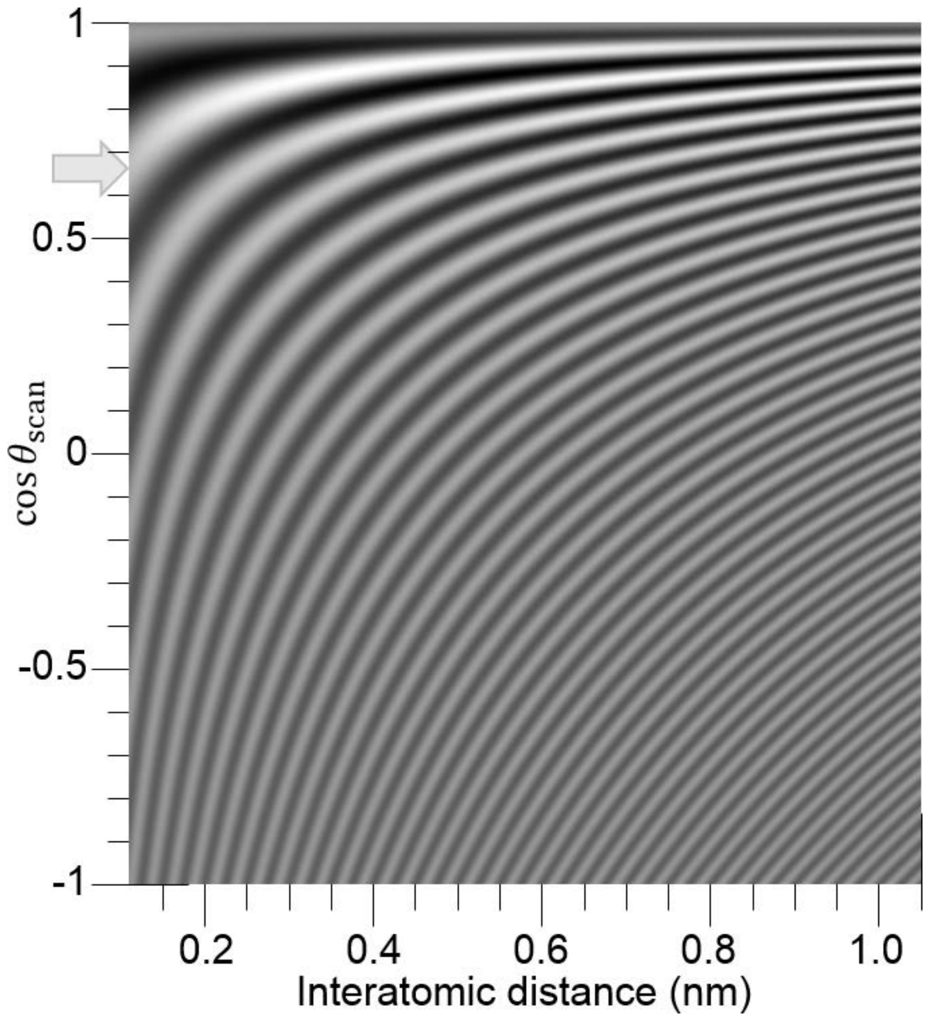

Fig.S4: Scattering pattern matrix for C at the kinetic energy of 600 eV. White and black contrast indicate construction and destruction interference of diffraction intensity. Arrows on the left side image shows the position of the first interference ring.
